# CMR feature tracking in Kawasaki Disease convalescence

**DOI:** 10.1186/1532-429X-17-S1-P366

**Published:** 2015-02-03

**Authors:** Konstantinos Bratis, Pauline Hackmann, Nicholas Child, Sophie Mavrogeni, Thomas Krasemann, Tarique Hussain, Rene Botnar, Reza Razavi, Gerald F Greil

**Affiliations:** 1 King's College London, London, UK; 2 Onassis Cardiac Surgery Centre, Athens, Greece; 3 Evelina ChildreN Hospital, London, UK

## Background

Myocardial inflammation has been described as a global finding in the acute phase of Kawasaki Disease. Despite normal LV systolic function by routine functional measurements, reduced longitudinal strain (S) and strain rate (SR) have been detected by echocardiography in the acute phase, which may potentially predict late onset heart failure.

We aimed to determine whether left ventricular (LV) myocardial deformation indices can detect subclinical myocardial abnormalities in Kawasaki Disease (KD) convalescence. We hypothesized that subclinical myocardial abnormalities due to inflammation represent an early manifestation of the disease that persist in convalescence.

## Methods

**Figure 1:**
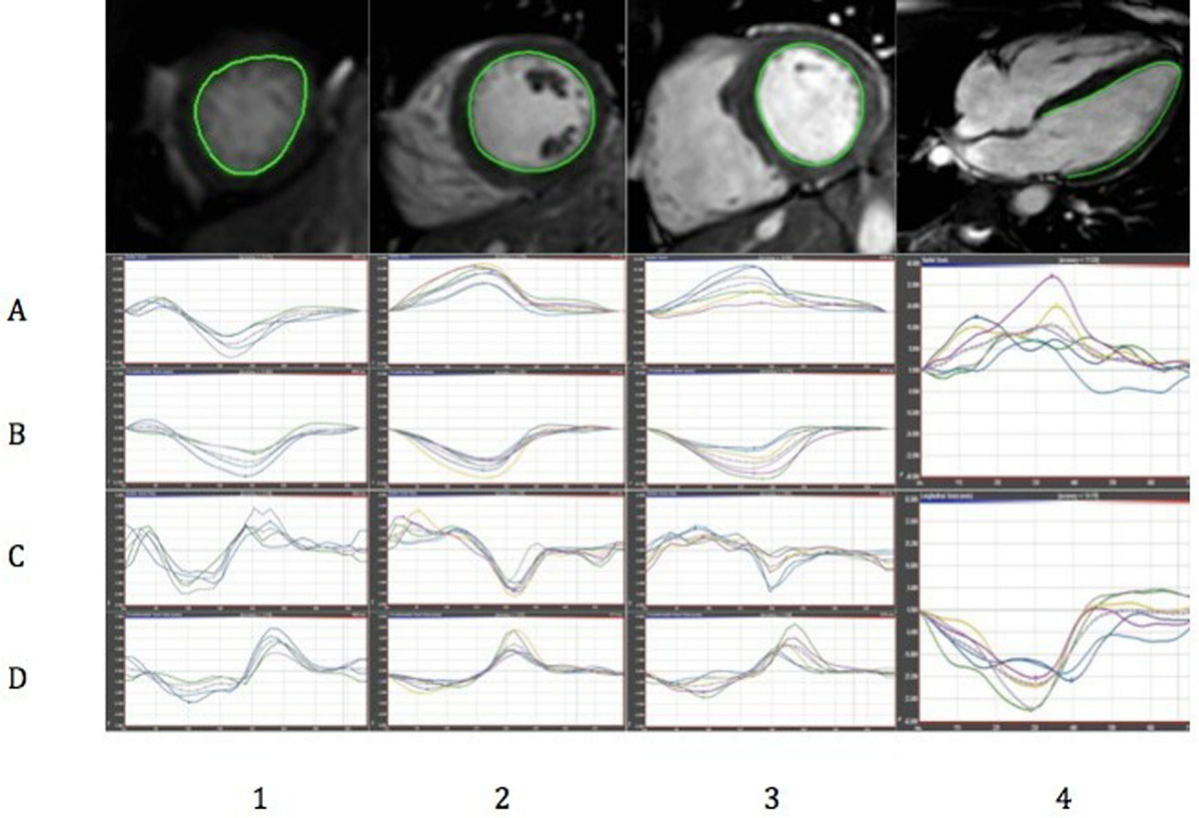
**CMR-FT post-processing.** Short-axis apical (1), mid ventricular (2), basal (3) and 4-chamber long-axis (4) views with relevant endocardial contour drawn in a KD patient. Radial strain (A) and strain rate (B), circumferential strain (C) and strain rate (D) and longitudinal strain and strain rate (4, mid and lower row, respectively) results are provided below each slice. CMR-FT: Cardiac Magnetic Resonance Feature Tracking, KD: Kawasaki Disease

Peak systolic LV myocardial longitudinal, radial and circumferential S and SR (Figure 1) were examined in 29 KD convalescent patients (15 males; mean (SD) age 11 (6.6) years, range 3- 27 years; median interval from KD onset 5.8 (5.4) years) and 10 healthy volunteers (5 males; mean age 14 (3.8) years, range 6- 19 years) with the use of cardiac magnetic resonance feature tracking (CMR-FT). Routine indices of LV systolic function were normal in both groups.

## Results

Comparisons were made between normal controls and (i) the entire KD group, (ii) KD group subdivided by coronary artery involvement. (Table 1) Compared to controls, KD patients had lower longitudinal S. Average longitudinal and circumferential S at all levels was lower in KD patients compared normal controls. In subgroup analysis, both KD patients with and without any history of CAD had similar longitudinal and circumferential S at all levels and lower when compared against controls. There was a non-significant trend for lower circumferential and longitudinal values in KD patients with persisting CAD when compared against those with regressed CAD.

**Table 1:** Longitudinal, radial and circumferential global LV deformation analysis of KD patients and KD subgroups compared with controls.

	Controls (n=10)	All KD (n=27)		KD with CAL		KD without CAL (n=5)
			All (n=22)	Persistent CAL (n=13)	Regressed CAL (n=9)	

Basal radial						

Strain	28.2 (13.9)	31.2 (8.3)	31.8 (8.6)	30.3 (8.0)	34.1 (9.0)	26 (3.2)

Strain rate	1.4 (0.4)	1.6 (0.7)	1.7 (0.8)	1.6 (0.9)	1.7 (0.6)	1.3 (0.2)

Basal circumferential						

Strain	28.2 (13.9)	23.7 (5.5)	23.1 (4.9)	22.0 (4.7)	24.9 (5.7)	23.1 (4.7)

Strain rate	1.4 (0.4)	1.5 (0.4)	1.5 (0.4)	1.5 (0.5)	1.5 (0.4)	1.3 (0.3)

Mid radial						

Strain	29.0 (7.3)	30.5 (8.1)	30.1 (5.3)	31.1 (5.2)	31.9 (11.4)	28.2 (7.7)

Strain rate	1.6 (0.2)	1.7 (0.8)	1.7 (0.8)	1.7 (1.0)	1.7 (0.5)	1.3 (0.3)

Mid circumferential						

Strain	23.8 (2.8)	21.1 (5.5)	21.0 (5.4)	20.9 (4.1)	22.2 (7.0)	21.9 (6.9)

Strain rate	1.7 (0.3)	1.4 (0.5)	1.4 (0.5)	1.4 (0.4)	1.5 (0.6)	1.3 (0.5)

Apical radial						

Strain	11.7 (2.8)	14.9 (7.4)	16.3 (6.0)	15.6 (6.3)	17.3 (5.7)	17.0 (8.6)

Strain rate	1.8 (0.7)	1.4 (0.7)	1.4 (0.6)	1.5 (0.8)	1.4 (0.3)	1.3 (0.8)

Apical circumferential						

Strain	24.9 (6.2)	21.7 (7.0)	22.0 (7.4)	21.0 (7.6)	24.5 (8.5)	17.4 (4.0)

Strain rate	1.9 (0.6)	1.6 (0.6)	1.6 (0.6)	1.6 (0.6)	1.8 (0.7)	1.4 (0.1)

Longitudinal						

Strain	18.9 (7.8)	16.4 (5.4)	17.2 (4.9)	16.2 (4.5)	18.8 (5.4)	12.8 (6.8)

KD: Kawasaki Disease, CAL: Coronary Artery Lesion, *: p < 0.05

## Conclusions

In this CMR-FT study in KD convalescent patients with preserved conventional functional indices, we observed a trend for lower circumferential and longitudinal strain in KD patients compared to normal controls, irrespective of their coronary artery status.

## Funding

The authors acknowledge financial support from the Department of Health through the National Institute for Health Research (NIHR) comprehensive Biomedical Research Centre award to Guy's & St Thomas' NHS Foundation Trust in partnership with King's College London and King's College Hospital NHS Foundation Trust. The Division of Imaging Sciences receives also support as the Centre of Excellence in Medical Engineering (funded by the Welcome Trust and EPSRC; grant number WT 088641/Z/09/Z) as well as the BHF Centre of Excellence (British Heart Foundation award RE/08/03).

